# Supplementary figures and images for: Comprehensive Steroid Assay with Non-Targeted Analysis Using Liquid Chromatography Ion Mobility Mass Spectrometry
Source: Int J Mol Sci. 2022 Nov 10;23(22):13858. doi: 10.3390/ijms232213858 (PMC9697045; doi:10.3390/ijms232213858)

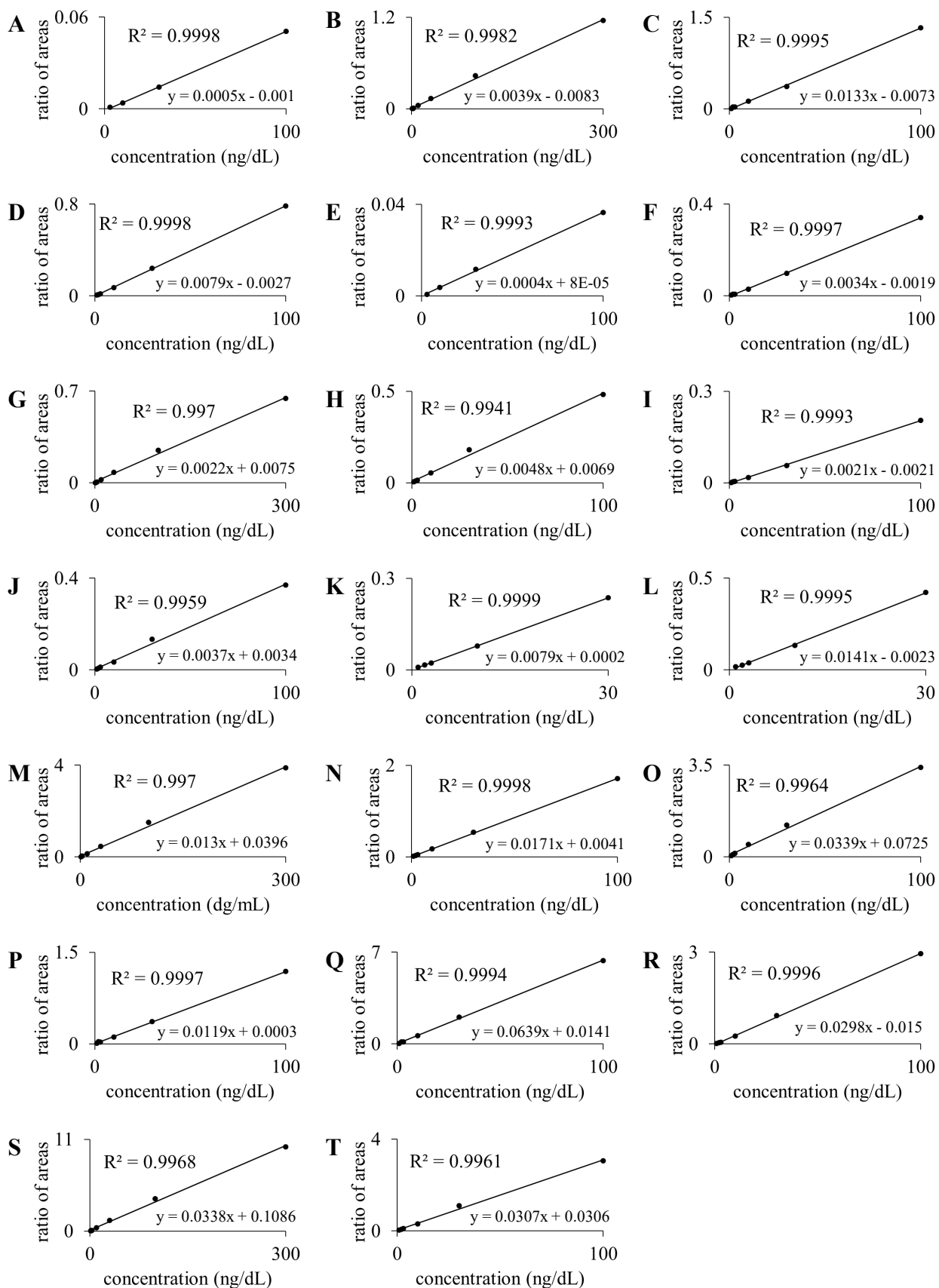

Supplementary Figure S1

Supplement: Supplementary file 1 [file ijms-23-13858-s001.zip › ijms-1978854-supplementary.pdf]
